# Near-Infrared Organic Phototransistors with Polymeric Channel/Dielectric/Sensing Triple Layers

**DOI:** 10.3390/mi11121061

**Published:** 2020-11-30

**Authors:** Taehoon Kim, Chulyeon Lee, Youngkyoo Kim

**Affiliations:** Organic Nanoelectronics Laboratory, KNU Institute for Nanophotonics Applications (KINPA), Department of Chemical Engineering, School of Applied Chemical Engineering, Kyungpook National University, Daegu 41566, Korea; k2557497@hanmail.net (T.K.); lcyyeon@naver.com (C.L.)

**Keywords:** near infrared, organic phototransistors, conjugated polymers, dielectric layers, photocurrent, threshold voltage, on/off modulation, LiDAR

## Abstract

A new type of near-infrared (NIR)-sensing organic phototransistor (OPTR) was designed and fabricated by employing a channel/dielectric/sensing (CDS) triple layer structure. The CDS structures were prepared by inserting poly(methyl methacrylate) (PMMA) dielectric layers (DLs) between poly(3-hexylthiophene) (P3HT) channel layers and poly[{2,5-bis-(2-octyldodecyl)-3,6-bis-(thien-2-yl)-pyrrolo[3,4-c]pyrrole-1,4-diyl}-co-{2,2′-(2,1,3-benzothiadiazole)-5,5′-diyl}] (PODTPPD-BT) top sensing layers. Two different thicknesses of PMMA DLs (20 nm and 50 nm) were applied to understand the effect of DL thickness on the sensing performance of devices. Results showed that the NIR-OPTRs with the CDS structures were operated in a typical n-channel mode with a hole mobility of ca. 0.7~3.2 × 10^−4^ cm^2^/Vs in the dark and delivered gradually increased photocurrents upon illumination with an NIR light (905 nm). As the NIR light intensity increased, the threshold voltage was noticeably shifted, and the resulting transfer curves showed a saturation tendency in terms of curve shape. The operation of the NIR-OPTRs with the CDS structures was explained by the sensing mechanism that the excitons generated in the PODTPPD-BT top sensing layers could induce charges (holes) in the P3HT channel layers via the PMMA DLs. The optically modulated and reflected NIR light could be successfully detected by the present NIR-OPTRs with the CDS structures.

## 1. Introduction

Organic phototransistors (OPTRs) have recently attracted considerable interest because they are capable of delivering flexible and wearable photosensor modules for various applications in the rapidly growing flexible electronics era [1,2,3,4,5,6,7]. Compared to inorganic photosensors (phototransistors), OPTRs have an advantage of low-cost fabrication when it comes to their wet-coating processes at room temperature [8,9,10,11,12]. Due to the three-electrode geometry, the photosensitivity of OPTRs can be controlled and amplified by adjusting the gate voltage (V_G_) between the source and gate electrodes in the devices [13,14,15,16,17].

Particular attention has been very recently paid to the near-infrared (NIR) light-sensing OPTRs since the NIR technology has become one of the most important cores for advanced control and sensing systems such as night vision for cars and airplanes, light detection and ranging (LiDAR) sensors for autonomous cars and drones, probe beams for biomedical devices and diagnostics, and optical communications. [18,19,20,21,22,23,24]. However, the NIR-absorbing organic materials are very rare because of the difficulty in synthesis to meet the narrow energy band (level) gap of ca. 0.89~1.65 eV, which corresponds to the wavelength (λ) range of ca. 750~1400 nm [25,26,27,28,29,30,31]. In this regard, conjugated polymers have been considered a viable chemical platform for the NIR-absorbing materials since their energy band gaps can be narrowed by combinations of electron-donating and electron-accepting comonomers [32,33,34,35,36,37,38].

In the basic structure of OPTRs, which is actually identical to organic field-effect transistors (OFETs), the channel layers should play a sensing role simultaneously [1]. However, some conjugated polymers do not have sufficient charge carrier mobility for the operation of OFETs even though they deliver good NIR-absorbing characteristics [39]. On this account, the NIR-absorbing conjugated polymers have been applied as a gate-sensing layer (GSL) in the advanced structure of OPTRs [40]. It is considered that the GSL concept could expand the choice of NIR-absorbing organic materials, regardless of whether they are semiconductors or not. Our recent work has demonstrated that the OPTRs with the GSL structure can be properly operated by applying the top channel layers with a sensing efficiency of 40~60% (λ = 780~1000 nm) compared to the theoretical maximum photoresponsivity [41]. However, further progress in device design on a microscale and/or nanoscale is required for the advancement of NIR-OPTRs which can be applied for various system environments [42,43,44,45].

Here, we demonstrate a new type of NIR-OPTR which consists of polymeric channel/dielectric/sensing (CDS) triple layers in the transistor geometry of bottom-gate and bottom- source/drain contact. The CDS structure was prepared by sequential spin-coating processes of poly(3-hexylthiophene) (P3HT), poly(methyl methacrylate) (PMMA), and poly[{2,5-bis-(2-octyldodecyl)-3,6-bis-(thien-2-yl)-pyrrolo[3,4-c]pyrrole-1,4-diyl}-co-{2,2′-(2,1,3-benzothiadiazole)-5,5′-diyl}] (PODTPPD-BT) on the silver electrode-deposited PMMA gate-insulating layers. The PMMA dielectric layer (DL) in the middle of the CDS structure was designed to play a dual role for the protection of beneath channel layers upon spin-coating as well as the dipole induction by photogenerated excitons (charges). To investigate the influence of PMMA DLs on the sensing performances, two different DL thicknesses (20 and 50 nm) were employed for the fabrication of the CDS structures. For practical applications, the sensing performances of the NIR-OPTRs with the CDS structures were examined under on/off modulation of NIR light and for the reflected (scattered) NIR light from an object.

## 2. Materials and Methods

### 2.1. Materials and Solutions

The P3HT polymer (weight-average molecular weight = 50 kDa) was purchased from Solaris Chem (USA). The PODTPPD-BT polymer (weight-average molecular weight = 8.7 kDa, polydispersity index (PDI) = 1.34) was synthesized via Suzuki coupling reaction using a palladium catalyst as reported in our previous work [41]. The P3HT solutions were prepared by employing toluene (Sigma-Aldrich, St. Louis, MO, USA) as a solvent at a solid concentration of 26 mg/mL, while the PODTPPD-BT solutions were prepared using chlorobenzene (Sigma-Aldrich, USA) at a solid concentration of 15 mg/mL. The PMMA polymer (weight-average molecular weight = 120 kDa) was purchased from Sigma-Aldrich (USA). For the preparation of gate-insulating layers, the PMMA solutions (80 mg/mL) were made using chlorobenzene as a solvent. To form the dielectric layers (DLs), n-butyl acetate was used as a solvent for the PMMA solutions with two different concentrations (3.6 and 9 mg/mL). All solutions prepared were subjected to continuous stirring on a hot plate at 60 °C for 24 h.

### 2.2. Thin Film and Device Fabrication

The OFETs with a bottom-gate–bottom-source/drain contact structure were fabricated using the patterned indium–tin–oxide (ITO)-coated glass substrates (ca. 20 Ω/cm^2^). The ITO–glass substrates were immersed in acetone and underwent ultrasonication processes for 30 min. Then the initially cleaned ITO–glass substrates were cleaned and rinsed with isopropyl alcohol using the same ultrasonic cleaner, followed by drying with nitrogen gas flow. The dried ITO–glass substrates were subjected to the 20 min treatment of ultraviolet (UV)/ozone using a UV/ozone cleaner (50 mW/cm^2^, AC-6, AHTECH LTS Co., Ltd., Anyang-si, Gyeonggi-do, Korea). On top of the ITO-coated sides of the treated substrates, the PMMA solutions (solvent: chlorobenzene) were spun at 2000 rpm for 60 s, leading to a 450 nm-thick gate-insulating layer. The PMMA layer-coated ITO–glass substrates were thermally treated at 120 °C for 60 min. After transferring these samples to a vacuum chamber, which is equipped inside an argon-filled glovebox, the 60 nm-thick silver (Ag) source/drain electrodes were deposited on the PMMA layers by thermal evaporation processes. Note that a shadow mask leading to a channel length of 70 µm and channel width of 2 mm was used during the deposition of Ag electrodes. The Ag electrode-deposited samples were moved out and thermally treated at 120 °C for 30 min. Next, the P3HT solutions were spun on top of the Ag electrode-deposited samples at 1500 rpm for 30 s, followed by soft-baking at 120 °C for 30 min. To form DLs, the two PMMA solutions (solvent: n-butyl acetate) were spun on the P3HT layers at 2000 rpm for 60s and then soft-baked 120 °C for 30 min. The thickness of the PMMA DLs was 20 nm and 50 nm for 3.6 mg/mL and 9 mg/mL, respectively. Finally, The PODTPPD-BT layers were formed on the PMMA DLs by dropping the PODTPPD-BT solutions upon spinning the DL-coated sample substrates at 1500 rpm for 60 s. The resulting thickness of the PODTPPD-BT layers was 50 nm. All devices fabricated were stored inside the argon-filled glovebox before measurement to minimize a possible attack of moisture and oxygen.

### 2.3. Measurement

A surface profilometer (Dektak XT, Bruker, Billerica, MA, USA) was used for the measurement of film thickness. A UV–visible-NIR spectrometer (Lambda 750, PerkinElmer, Waltham, MA, USA) was employed to measure the optical absorption spectra of film samples. The channel area of OFETs was examined on a microscale using an optical microscope (SV-55, Sometech, Seoul, Korea). The transistor performances were measured using a semiconductor parameter analyzer (2636B, Keithley, Cleveland, OH, USA). For the measurement of phototransistor performances, the channel area of devices was illuminated with a laser diode (905 nm, VD9030V, Delos Laser, Seoul, Korea). All devices and laser diodes were placed inside a dark metal box to avoid any influence by ambient light. The incident light intensity (P_IN_) of the laser diode was measured using a calibrated photodiode (818-UV, Newport, Irvine, CA, USA), while it was adjusted using a neutral density filter set (CVI Melles-Griot SP Pte. Ltd., Singapore). For the measurement of reflected and scattered NIR light, the 905 nm light from the laser diode was irradiated to an object (optical post holder, PH-3, NAMIL Optical Instrument Co.), and the resulting scattered light was detected by the OPTRs fabricated in this work.

## 3. Results and Discussion

**Figure 1:**
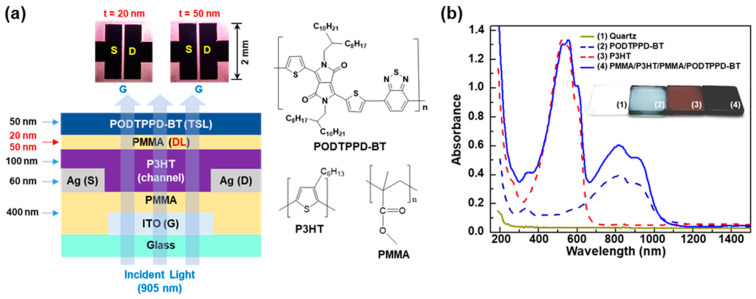
(**a**) Illustration of the cross-sectional structure of the device (see chemical structures on the right) and optical microscope images of the channel part for the near-infrared-sensing organic phototransistors (NIR-OPTRs) with the channel/dielectric/sensing (CDS) structures consisting of poly(3-hexylthiophene) (P3HT)/poly(methyl methacrylate) (PMMA)/poly[{2,5-bis-(2-octyldodecyl)-3,6-bis-(thien-2-yl)-pyrrolo[3,4-c]pyrrole-1,4-diyl}-co-{2,2′-(2,1,3-benzothiadiazole)-5,5′-diyl}] (PODTPPD-BT) triple layers (note that two different thicknesses (t = 20 nm and t = 50 nm) were applied for the PMMA dielectric layers (DLs)). (**b**) Optical absorption spectra of films coated on quartz (1) substrates: (2) PODTPPD-BT (50 nm), (2) P3HT (100 nm), (3) PMMA/P3HT/PMMA/PODTPPD-BT (CDS). Inset photographs show the color difference of films.

As illustrated in Figure 1a, the present OPTRs feature polymeric channel/dielectric/sensing (CDS) triple layers which are composed of the multi-stacked P3HT/PMMA/PODTPPD-BT polymers. The P3HT channel layers can be protected by the PMMA dielectric layers from the solvent attack upon spin-coating using the PODTPPD-BT solution (solvent: CB) for the preparation of the top sensing layers. Note that PMMA was dissolved in n-butyl acetate only at a high temperature (>70 °C) and n-butyl acetate did not dissolve the P3HT layers at all. Similarly, the thick (450 nm) PMMA gate-insulating layers were found not to be seriously affected by the CB solvent when it comes to the short spin-coating time (30 s) for the preparation of the P3HT channel layers. In order to minimize any possible influence on the very thin (20~50 nm) PMMA DLs, the PODTPPD-BT solutions were dropped when spinning the PMMA DL-coated samples so that the contact time of CB solvent could be as short as 1 or 2 s. The resulting CDS structures were prepared on quartz substrates for the examination of optical absorption properties. As shown in Figure 1b, the prepared CDS structures (P3HT/PMMA/PODTPPD-BT) delivered a broadband absorption covering whole visible light and the NIR region up to 1100 nm even though the pristine P3HT and PODTPPD-BT layers could absorb limited visible and NIR regions, respectively. The inset photographs in Figure 1b provide eye-catching evidences for the well-prepared CDS structures. This result confirms that the PMMA DLs did successfully play a role in protecting the P3HT channel layers from the CB solvent upon spin-coating of the PODTPPD-BT top sensing layers.

**Figure 2:**
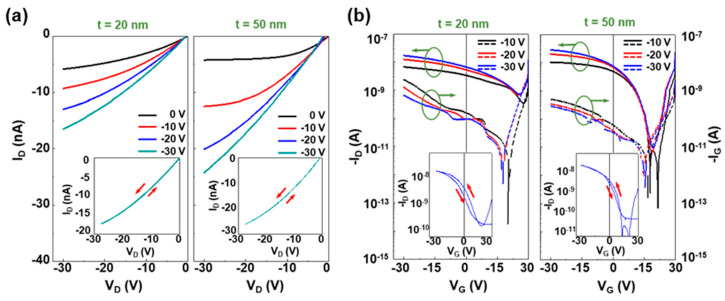
(**a**) Output and (**b**) transfer curves (left: drain current, right: gate current) of the NIR-OPTRs with the CDS structures in the dark according to the thickness (t) of PMMA DLs (left: t = 20 nm, right: t = 50 nm). The inset graphs in (a) show the representative output curves (swept between V_D_ = 0 V and V_D_ = −30 V) at V_G_ = −30 V, while those in (b) show the representative transfer curves (swept between V_G_ = 0 V and V_G_ = −30 V) at V_D_ = −30 V.

The performances of the present OPTRs in the dark were measured to understand the basic characteristics of the transistors with the CDS structures. As observed from the output curves in Figure 2a, the devices showed a typical p-channel transistor behavior with a clear dependency of drain current (I_D_) on the gate voltage (V_G_) at a fixed drain voltage (V_D_). Here, interestingly, the level of drain current was relatively lower for the devices with the 20 nm-thick PMMA DLs than those with the 50 nm-thick DLs. A similar trend of drain current difference was measured for the transfer curves (see Figure 2b). The sweeping test in the dark unveiled almost no hysteresis in the output curves but very slight hysteresis in the transfer curves (see the inset graphs in Figure 2). In addition, the off current was considerably poor for the devices with the 20 nm-thick PMMA DLs compared to the devices with the 50 nm-thick DLs (refer to the gate current (I_G_) for each case).

The poor dark performances of the devices with the 20 nm-thick PMMA DLs can be attributed to the imperfect protection role of the 20 nm-thick DLs against the attack of CB solvent during the PODTPPD-BT top sensing layers when it comes to the higher possibility of pinhole generation in a thinner film than a thicker film. In more detail, some parts of the CB solvents might permeate through pinholes in the 20 nm-thick DLs and cause partial damage to the P3HT channel layers, leading to such a poor device performance. From the I_D_^0.5^-V_G_ curves, the hole mobility (μ_h_) of the present OPTRs in the dark was calculated as ~0.7 × 10^−4^ cm^2^/Vs and ~3.2 × 10^−4^ cm^2^/Vs for the 20 nm-thick and 50 nm-thick PMMA DLs, respectively (see Table 1). The high threshold voltage (V_TH_) toward a positive voltage direction may reflect the existence of interfacial charges formed in due course of multi-layer deposition processes in the present device structures [46].

**Figure 3:**
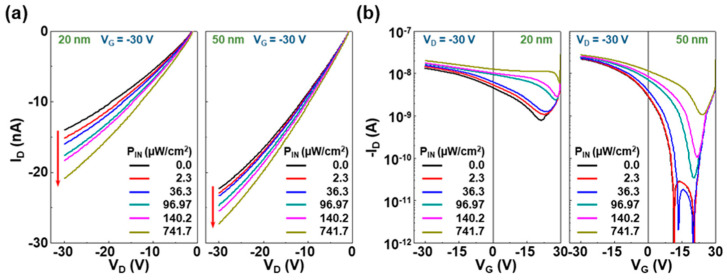
(**a**) Output and (**b**) transfer curves for the NIR-OPTRs with the CDS structures under illumination with the NIR light (905 nm). The incident NIR light intensity (P_IN_) was varied between 2.3 and 742 mW/cm^2^.

Next, the OPTRs with the 20 nm-thick and 50 nm-thick PMMA DLs were subjected to the examination of photosensing characteristics under illumination with NIR light using a high-power laser diode used for practical LiDAR applications (wavelength (λ) = 905 nm). First, the NIR sensing characteristics were measured by adjusting the output of laser diode to a lower light density level of ca. 2.3~742 μW/cm^2^. As shown in Figure 3a, for both 20 nm-thick and 50 nm-thick PMMA DLs, the output curves at V_G_ = −30 V were gradually shifted toward a (negatively) higher drain current direction with increasing the incident NIR intensity (P_IN_). This result basically informs that the present OPTRs with the CDS structures did properly work and respond to the incident NIR light. Because the P3HT channel layers and PMMA layers do not absorb any NIR light, it is obvious that the PODTPPD-BT top sensing layers should absorb the incident NIR light, and the generated excitons might act as a floating gate (external bias) to induce charges in the P3HT channel layers via the PMMA DLs. A similar working mechanism has been reported by applying liquid crystals (LCs) with a high dielectric constant in our previous reports [46,47,48]. Here, it is also worth noting that the drain current difference became larger at the higher drain voltage. This may directly reflect that more charges (holes) in the P3HT channel layers, which were induced by the photo-generated excitons in the PODTPPD-BT top sensing layers, could be transported at higher drain voltages. Further investigation into the transfer curves at V_D_ = −30 V finds that there were gradual shifts in threshold voltages with the incident NIR light intensity (see Figure 3b). Note that the intrinsic off-current characteristics were not changed upon the NIR light illumination as the poor off current of the devices with the 20 nm-thick PMMA DLs was kept for all P_IN_ cases. Considering that the threshold voltage shift does in principle indicate the charge trapping phenomena in devices, it is supposed that the charges induced from the excitons generated in the PODTPPD-BT top sensing layers might be trapped at the layer interfaces of the CDS structures. The threshold voltage shift toward a positive voltage reflects that the trapped charges in the CDS structure did mainly induce holes in the P3HT channel layers.

**Figure 4:**
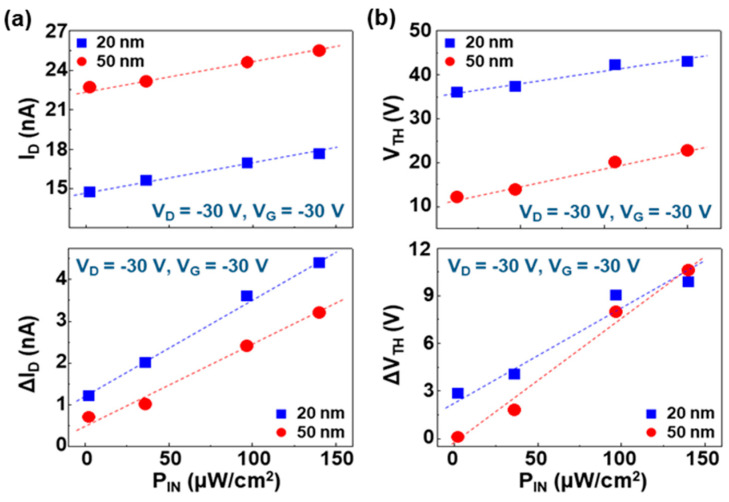
Variation of sensing parameters (taken from the transfer curves in Figure 3) as a function of the incident low-power NIR light intensity (P_IN_ = ca. 2.3~140.2 μW/cm^2^): (**a**) drain current (I_D_) and net drain current (ΔI_D_), (**b**) threshold voltage (V_TH_) and threshold voltage shift (ΔV_TH_). The slope (ΔV_TH_/P_IN_) was 6.22 × 10^4^ V·cm^2^/W (t = 20 nm) and 7.63 × 10^4^ V·cm^2^/W (t = 50 nm).

To understand the detailed trend, the device parameters were plotted as a function of the incident NIR light intensity. As shown in Figure 4a top panel, the overall drain current was almost linearly increased with the incident NIR light intensity for both cases (20 nm-thick and 50 nm-thick PMMA DLs). After removing the dark current portion, the linearity was still kept with the incident NIR light intensity (see Figure 4a bottom panel). This result implies that the similar portion (ratio) of charges induced by the excitons generated in the PODTPPD-BT top sensing layers was transported between the source and drain electrodes irrespective of the incident NIR light intensity. Taking into account the linearly increasing trend of threshold voltage (see Figure 4b top panel), the ratio of trapped charges might be proportionally increased with the incident NIR light intensity for both devices. However, a close look into the slopes of net photocurrent (ΔI_D_) as well as net threshold voltage shift (ΔV_TH_) may deliver that, as the incident NIR light intensity increased, the ratio of charge transport to charge trap became higher for the OPTRs with the thinner (20 nm) PMMA DLs. In other words, more charges could be trapped for the OPTRs with the thinner (20 nm) PMMA DLs at higher incident NIR light intensity. This result can give a rough clue regarding the degree of film perfectness (vice versa, pinhole-like defects) in the two different thicknesses of PMMA DLs. Given that, over the whole range of incident NIR light intensity, the net photocurrent was always higher for the OPTRs with the thinner PMMA DLs than those with the thicker DLs (see Figure 4a bottom panel), the charges induced via the DLs from the excitons generated in the PODTPPD-BT top sensing layers might follow the basic relation of capacitance–thickness that defines higher capacitances at lower thicknesses [49].

**Figure 5:**
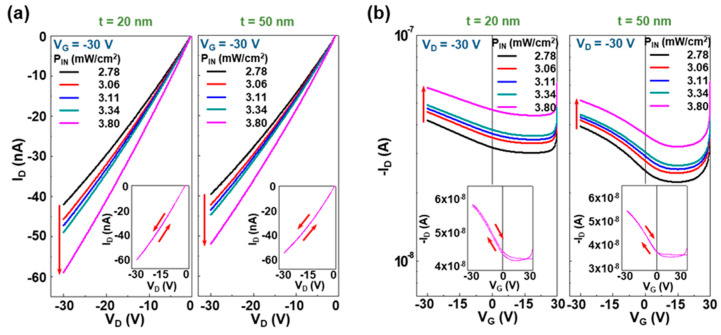
(**a**) Output and (**b**) transfer curves for the NIR-OPTRs with the CDS structures under illumination with the NIR light (905 nm). The incident NIR light intensity (P_IN_) varied between 2.8 and 3.8 mW/cm^2^. The inset graphs in (a) show the representative output curves (swept between V_D_ = 0 V and V_D_ = −30 V) at V_G_ = −30 V and P_IN_ = 3.80 mW/cm^2^, while those in (**b**) show the representative transfer curves (swept between V_G_ = 0 V and V_G_ = −30 V) at V_D_ = −30 V and P_IN_ = 3.80 mW/cm^2^.

For the practical applications, a stronger NIR light with a power density of ca. 2.8~3.8 mW/cm^2^ was exposed to the present OPTRs with the CDS structures. As shown in Figure 5a, the output curves were largely shifted with the incident NIR intensity for both devices. In addition, the higher drain current difference was measured at the higher drain voltage, which was similarly observed in the case of low-power NIR irradiations in Figure 3. This result supports the notion that the present OPTRs with the CDS structures function properly for the detection of high-power NIR light as well. Note that the drain current measured at P_IN_ = 3.8 mW/cm^2^ was still higher for the OPTRs with the 20 nm-thick PMMA DLs than those with the 50 nm-thick DLs. In particular, all the transfer curves in Figure 5b showed a largely different shape from those in Figure 3b, as the drain current in the positive voltage region was not steeply dropped. This can be ascribed to the considerably shifted threshold voltages by the trapped charges under the high-power NIR light illumination. As the incident NIR intensity increased from P_IN_ = 2.8 mW/cm^2^ to P_IN_ = 3.8 mW/cm^2^, the transfer curves were gradually shifted toward a higher drain current direction. Note that the sweeping test revealed almost no hysteresis in the output curves but very slight hysteresis in the transfer curves (see the inset graphs in Figure 5).

**Figure 6:**
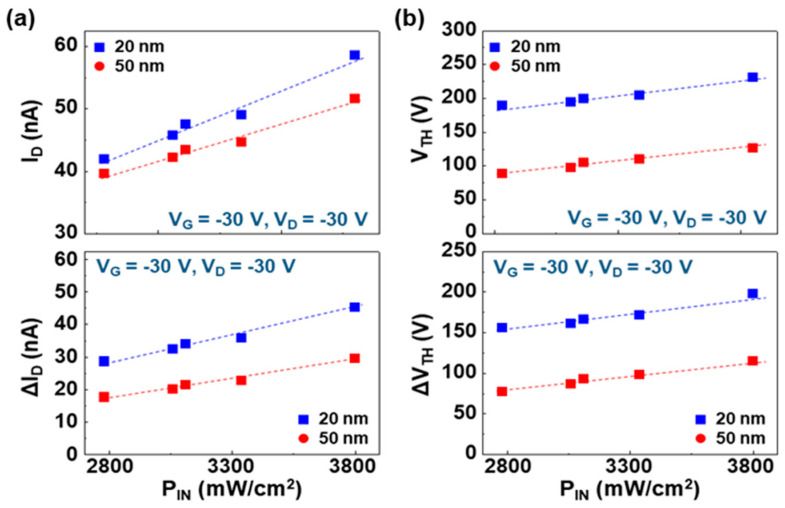
Variation of sensing parameters (taken from the transfer curves in Figure 5) as a function of the incident high-power NIR light intensity (P_IN_ = 2.8~3.8 mW/cm^2^): (**a**) drain current (I_D_) and net drain current (ΔI_D_), (**b**) threshold voltage (V_TH_) and threshold voltage shift (ΔV_TH_). The slope (ΔV_TH_/P_IN_) was 4.11 × 10^4^ V·cm^2^/W (t = 20 nm) and 3.7 × 10^4^ V·cm^2^/W (t = 50 nm).

The detailed changes of drain current and threshold voltage were analyzed and plotted as a function of the high-power incident NIR intensity. As shown in Figure 6a top panel, the overall drain current showed a linearly increasing trend with the high-power incident NIR intensity between 2.8 mW/cm^2^ and P_IN_ = 3.8 mW/cm^2^. The net photocurrent (ΔI_D_) was linearly increased with the high-power incident NIR intensity irrespective of the thickness of DLs (see Figure 6a bottom panel), while the increasing slope was slightly higher for the OPTRs with the 20 nm-thick PMMA DLs than those with the 50 nm-thick PMMA DLs. This trend was in accordance with the result in Figure 4a. However, as observed from Figure 6b, the slope of threshold voltages was almost similar for both devices, which is different from the result in Figure 4b. Therefore, it is considered that the degree of threshold voltage shift might be less sensitive to the incident NIR intensity in the case of a high-power regime because the interfaces and/or channels could be considerably saturated by the induced charges (holes) due to the high population of excitons generated in the PODTPPD-BT top sensing layers. Here, it is worth noting that the net threshold shift (ΔV_TH_) was still higher for the OPTRs with the 20 nm-thick PMMA DLs than those with the 50 nm-thick PMMA DLs.

**Figure 7:**
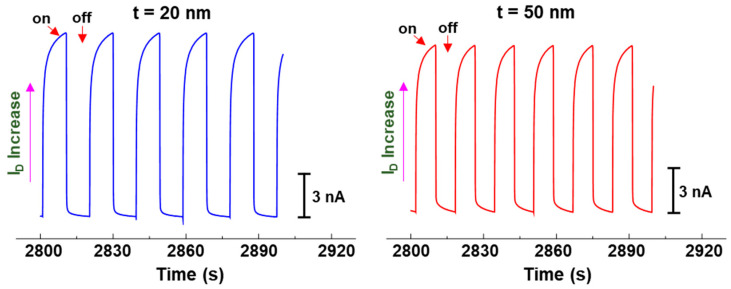
Drain current change with time upon illumination with the optically modulated NIR light (P_IN_ = 0.695 mW/cm^2^, 905 nm) for the optimized NIR-OPTRs with the CDS structures at V_D_ = −30 V and V_G_ = −30 V: (**left**) t = 20 nm, (**right**) t = 50 nm.

Finally, the present OPTRs with the CDS structures were tested for the direct or indirect detection of NIR (905 nm) light that is optically modulated with a constant on/off frequency. As shown in Figure 7, the drain current was quickly increased when the modulated NIR light was incident to the OPTRs irrespective of the thickness of DLs. However, there was a delayed increase after the initial quick jump for both devices, which can be attributed to the charging behavior of devices when the OPTRs were exposed to the NIR light for such a long time. When the NIR light was blocked (off phase in modulation), the drain current was quickly dropped in the presence of a marginal drain current tail. The slope of the tail drain current signals was slightly higher for the OPTRs with the 50 nm-thick PMMA DLs than those with the 20 nm-thick PMMA DLs, indicative of more charge trapping behavior in the thicker PMMA DLs.

**Figure 8:**
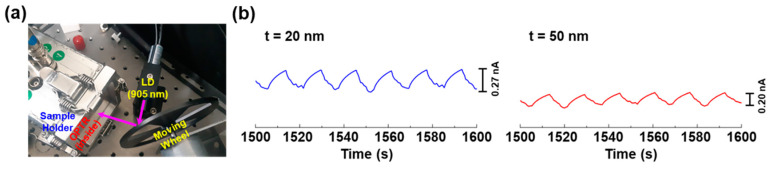
Sensing test for the reflected (scattered) NIR light from an object (moving wheel) using the optimized NIR-OPTRs with the CDS structures: (**a**) photograph for the measurement setup in which the NIR-OPTR is mounted inside the sample holder; (**b**) drain current signals from the NIR-OPTR upon slow moving of the wheel. The initial light intensity from the 905 nm laser diode (LD) was ca. 3.8 mW/cm^2^, while the applied drain and gate voltages were V_D_ = −30 V and V_G_ = −30 V, respectively.

As illustrated in Figure 8a, the reflected NIR light was detected by the present OPTRs. When the moving wheel was slowly rotated, the NIR light was reflected or scattered by the wheel frame, and then some part of the reflected NIR light could be incident to the OPTR mounted inside the sample holder. As shown in Figure 8b, the OPTRs could successfully detect the reflected NIR light irrespective of the thickness of PMMA DLs. This result implicates that the present OPTRs can be potentially used as an actual sensor for the LiDAR systems which should properly detect a reflected NIR light from an object [22].

## 4. Conclusions

The NIR-OPTRs with the channel/dielectric/sensing (CDS) triple layers were successfully fabricated by applying two different thicknesses of the PMMA DLs. The devices with the CDS structures showed typical p-channel transistor performances in the dark irrespective of the DL thickness, while their hole mobility was measured in the range of 0.7~3.2 × 10^−4^ cm^2^/Vs. Upon illumination with the low-power NIR light (905 nm), the drain current of devices was gradually increased with the NIR light intensity in both output and transfer curves. In addition, the threshold voltage in the transfer curves was shifted proportionally with the intensity of the low-power NIR light. The similar gradual drain current increase was measured upon illumination with the higher power NIR light, while the shape of transfer curves was almost identical for the NIR-OPTRs with the same DL thickness. The net photocurrent was higher for the NIR-OPTRs with the 20 nm-thick DLs than those with the 50 nm-thick DLs, which can be explained by the basic capacitance–thickness relation defining higher capacitances at lower thicknesses. These results confirm that the CDS structures in the present devices do actually function as a sensing medium for NIR light via a charge induction mechanism that forms charges (holes) in the P3HT channel layers through the PMMA DLs from the excitons generated in the PODTPPD-BT top sensing layers. The optimized NIR-OPTRs with the CDS structures exhibited stable sensing performances upon on/off modulation of NIR light and could sense the reflected (scattered) NIR light from an object. This test supports that the present NIR-OPTRs with the CDS structures are promising as a potential NIR sensor for LiDAR systems.

## Figures and Tables

**Table 1:** Summary of parameters (dark condition) for the NIR-OPTRs with the CDS structures according to the thickness (t) of PMMA DLs. The data were extracted from the transfer curves in Figure 2b at V_G_ = −30 V, V_D_ = −30 V. I_D,max_ and R_ON/OFF_ denote the maximum drain current (V_G_ = −30 V, V_D_ = −30 V) and on/off ratio, respectively.

t (nm)	I_D,max_ (nA)	V_TH_ (V)	R_ON/OFF_	μ_h_ (10^−4^ cm^2^/V∙s)
20	17.7	36.7	23.8	0.7
50	28.5	17.5	2930	3.2
